# Comparison of rest redistribution and traditional set configurations in terms of strength, power, and perceived exertion: a systematic review and meta-analysis of randomized trials

**DOI:** 10.1186/s13102-026-01709-6

**Published:** 2026-04-24

**Authors:** Fatih Gür, Beste Serter

**Affiliations:** 1 Department of Movement and Training Sciences, Faculty of Sport Science, University of Pamukkale, Denizli, 20000 Turkey; 2 Institute of Health Sciences, Department of Movement and Training Sciences, University of Pamukkale, Denizli, Turkey

**Keywords:** Rest redistribution, Traditional set, Resistance training, Rest configurations, Perceived exertion

## Abstract

**Objective:**

The rest redistribution (RR) method is a current approach used in resistance training that distributes the same total duration as the traditional set (TS) method but in shorter and more frequent rest intervals. This systematic review and meta-analysis aim to bridge the existing gap by primarily comparing the effects of RR and TS configurations in resistance training, with a specific focus on strength, power-related performance outcomes, and perceived exertion.

**Methods:**

The review was conducted in accordance with the Preferred Reporting Items for Systematic Reviews and Meta-Analyses (PRISMA). The literature search encompassed the Web of Science, PubMed/MEDLINE, Scopus, and SPORTDiscus electronic databases and was last updated on 20 May 2025. Eligible studies included randomized controlled trials (both parallel and crossover designs) involving healthy, active individuals that directly compared RR and TS configurations during resistance training. Methodological quality was evaluated using the Cochrane Risk of Bias 2 tool.

**Results:**

Fifteen studies involving 346 participants were included. The RR set configuration resulted in significantly greater improvements in mean power (SMD = 0.39, 95% CI: 0.16 to 0.72) and peak torque (SMD = 0.23, 95% CI: 0.10 to 0.36) compared to TS. Additionally, RR was associated with significantly lower perceived exertion (SMD = -0.67, 95% CI: -1.04 to -0.30). No significant differences were observed between RR and TS for peak power, mean velocity, maximal force, or mean force.

**Conclusion:**

In conclusion, this meta-analysis compared the effects of RR and TS configurations on multiple strength- and power-related outcomes as well as perceived exertion. The findings indicate that RR provides significant advantages in mean power, peak torque, and perceived exertion, while no significant differences were observed in maximal force, mean force, peak power, or mean velocity. These results suggest that although RR may offer benefits for certain performance outcomes, its effects are not uniform across all strength and power variables.

**Trial registration:**

The study protocol for the present systematic review was preregistered in the PROSPERO database under registration number CRD42024496872. Available from: https://www.crd.york.ac.uk/PROSPERO/view/CRD42024496872.

**Supplementary Information:**

The online version contains supplementary material available at 10.1186/s13102-026-01709-6.

## Background

Resistance training is a type of exercise where force is generated against resistance obtained from free weights, machine weights, elastic bands, or body weight [1]. The literature extensively demonstrates the positive effects of resistance training on health and athletic performance parameters. Among these effects are the development of muscular fitness leading to increased functional capacity [2], a reduced risk of osteoporosis due to increased bone density [3] and regulation of resting heart rate and cholesterol levels contributing to improved cardiovascular health [4]. Additionally, in conjunction with resistance training, progress is observed in strength, balance, agility, and speed capacities [5]. It is well known, especially in young athletes, that athletic performance improves and the risk of injuries decreases [6, 7].

The effectiveness of resistance training is influenced by various factors such as the volume of training, rest time, intensity, and type of training [8, 9]. In recent years, one of the variables used to manipulate resistance training is the inter-set rest intervals [10]. In traditional resistance training, rest intervals are typically applied between sets rather than between repetitions, and this structure is referred to as the traditional set (TS) configuration. In contrast, cluster sets (CS) involve additional rest intervals within sets, often between repetitions, while rest redistribution (RR) reorganizes total rest time into more frequent but shorter intervals across a greater number of sets. In traditional resistance training, rest intervals are typically applied between sets, while repetitions within a set are performed consecutively without any programmed rest. This structure is referred to as the TS configuration. In contrast, methods that incorporate additional rest intervals within sets or between individual repetitions are known as CS [11]. An important disadvantage of CS training is that it extends the total session duration (i.e., the total time required to complete the training session) compared to the TS structure, due to the additional rest intervals inserted between repetitions. Another innovative set-restructuring method that addresses this disadvantage is the RR method [12]. In this approach, the TS structure is reorganized to have a greater number of sets with shorter rest intervals while keeping the total rest time constant [13].

Upon reviewing the literature, it is observed that CS and RR methods may provide advantages compared to TS structures in terms of features such as the formation of acute fatigue during repetitions and sets, maximum speed, and power production [14–16]. The underlying physiological mechanism behind this situation is suggested to involve the better preservation of phosphocreatine stores, an increased rate of adenosine triphosphate resynthesis, and more effective clearance of the increased metabolic load in the muscles when longer or more frequent rest intervals are provided during resistance training [17].

The RR technique has been the subject of several research studies, providing evidence of its effectiveness in various contexts. Tufano et al. [18] conducted a study comparing TS versus RR and found that RR appeared to be more efficient during high-velocity movements, indicating its potential effectiveness in specific exercise scenarios. However, this meta-analysis primarily includes studies that utilized traditional resistance exercises, such as back squats, rather than high-velocity movements. Additionally, Chae et al. [19] explored the effect of RR with heavier loads on physiological and perceptual responses in resistance trained men and showed that the RR method may be more effective than traditional sets for increasing volume load without increasing acute fatigue responses. Furthermore, Boffey et al. [20] suggested in their research on male and female participants that during RR, compared to TS, both the ability to maintain movement velocity was at a higher level and perceived effort levels were lower. On the other hand, Jukic and Tufano [21] reported in their research comparing the RR method with TS based approaches that there was no significant difference in average power and mean velocity between the two methods. However, they noted a significant difference in favor of RR concerning the perceived exertion levels. Similarly, Chae et al. [19] in their study reported a favorable outcome for the RR method in terms of force and velocity, but noted no significant difference in terms of power values between the two methods. Additionally, Oliver et al. [22] mentioned that training performed with TS based methods may lead to greater hypertrophic growth compared to the RR configuration.

As can be seen the evidence regarding the effectiveness of RR set configurations compared to TS configurations in resistance training is mixed. Despite the increasing interest in the RR method in recent years, just two meta-analysis study has been identified [16, 17], aiming to synthesize existing evidence. These two studies have investigated the acute and chronic effects of CS and RR methods compared to TS. On the other hand, the number of studies included in the analysis for RR is limited for some parameters. For instance, perceived exertion has been analyzed in only three studies, and mean velocity decrement has been examined in four studies [17]. Following this relevant research, it has been observed that a significant number of new research articles have been published on the subject [19, 20, 23–34].

By incorporating these new research findings, a systematic review and meta-analytical approach can contribute to the evaluation of the topic, providing practitioners and athletes with clear guidelines for optimizing resistance training protocols. This systematic review and meta-analysis aim to bridge the existing gap by primarily comparing the effects of RR and TS configurations in resistance training, with a specific focus on strength, power-related performance outcomes (e.g., mean power, peak power, and velocity), and perceived exertion.

## Methods

### Protocol and registration

The review was conducted in accordance with the Preferred Reporting Items for Systematic Reviews and Meta-Analyses (PRISMA) [35] (Supplementary File 1). The study protocol for the present systematic review was preregistered in the PROSPERO database under registration number CRD42024496872.

### Search strategy

A comprehensive literature search was conducted to identify relevant studies for inclusion in this meta-analysis. Electronic databases, including, Web of Science, PubMed/MEDLINE, Scopus, and SPORTDiscus, were systematically searched ensure a thorough coverage of the literature. The search strategy encompassed a combination of Medical Subject Headings (MeSH) and free text words, targeting key concepts pertaining to the influence of various recovery strategies employed in strength training. Additional terms such as “endurance” and “hypertrophy” were included to increase the sensitivity of the search strategy and to ensure that all potentially relevant studies examining resistance training adaptations under different set configurations were captured. The following index of keywords and their combinations were utilized during the search: (“rest redistribution*” or “rest loading” or “rest interval” or “rest period” or “cluster set*” or “traditional set” or “set configuration” or “inter set rest” or “intra-set rest”) and (“power” or “strength” or “resistance” or “force” or “endurance” or “exercise” or “training” or “weightlifting” or “fatigue” or “hypertrophy*” or “recovery”).

**Fig. 1:**
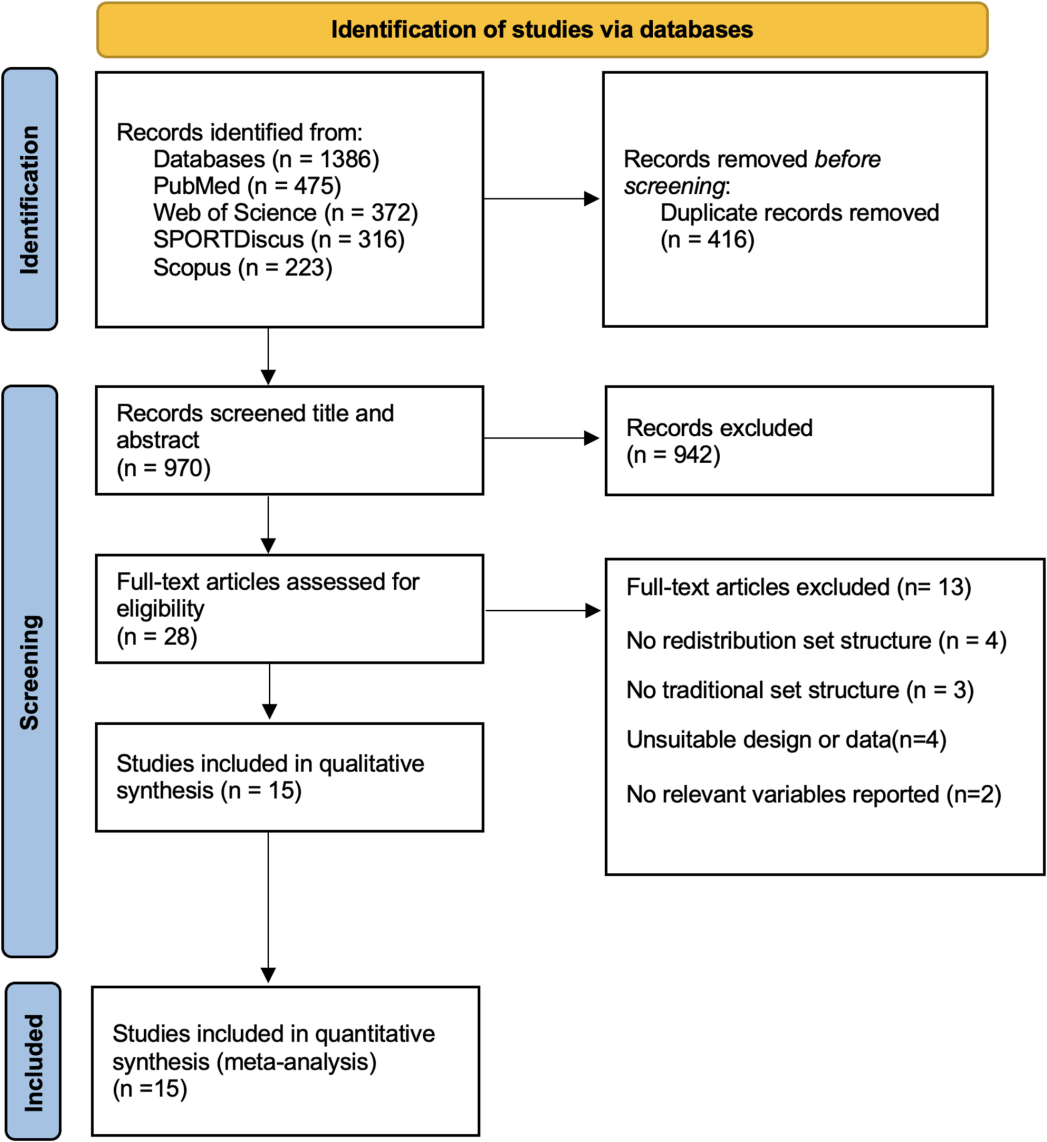
Flow chart for the search procedure

The search was performed from 20 May 2025, without imposing any publication year restrictions or filters. After removing duplicates, a two-stage search methodology was employed. Initially, titles and abstracts were evaluated to exclude irrelevant articles based on predetermined eligibility criteria. In the second stage, the full texts of the articles selected in the first stage were thoroughly assessed to determine their adherence to the inclusion criteria. Additionally, an extensive examination of the reference lists of retrieved papers, following the guidelines outlined by Greenhalgh and Peacock [36], was conducted to identify potential new studies meeting the inclusion criteria. To minimize selection bias, two researchers (F.G. and B.S.) independently performed the search and screening process. When disagreements arose regarding the inclusion of a particular study, the parties met to discuss and reach a final decision through consensus. Inter-rater agreement for the study selection process was high, with a Cohen’s kappa coefficient of 0.88, indicating excellent agreement between the two reviewers. The search methodology is visually represented in Fig. 1.

### Selection criteria

The analysis included studies that met the following PICOs criteria. Population (P): Healthy and physically active individuals who engage in resistance training. Intervention (I): RR method in resistance training. Comparison (C): Comparison of the TS and RR method in resistance training. Outcome (O): Strength, power, mean velocity and perceived exertion levels. The inclusion criteria of the study were determined as follows: The inclusion criteria for this meta-analysis encompassed studies published in peer-reviewed journals with a randomized trial (RT) design. Specifically, studies were considered eligible if they involved resistance training as the primary mode of exercise and compared the RR method and TS method in the context of resistance training. The RR method is an approach during resistance training with a total rest time similar to TS, but it involves a higher number of sets and shorter rest intervals. Studies that explored other rest period strategies such as intra-set rest, inter-repetition rest, and rest–pause models, were also considered, provided that total rest times were equal in both approaches. The focus was on studies reporting outcomes related to muscle strength gains, power, velocity, and perceived exertion levels. Only studies with available full-text articles or sufficient data for analysis were included. Conversely, studies focusing on populations with specific medical conditions or injuries that may affect muscle strength or power gains were excluded. Additionally, studies examining rest period strategies in combination with other interventions were not considered. Although these set configurations (e.g., intra-set rest, inter-repetition rest, and rest–pause) are not identical, they were grouped under the concept of rest redistribution, as they share a common underlying principle of distributing total rest time into shorter and more frequent intervals. This approach is consistent with previous literature examining clustered or redistributed rest structures. However, it is acknowledged that this conceptual grouping may introduce some degree of heterogeneity across studies.

### Data extraction

The data extraction process was conducted by the researchers using the Rayyan.ai website (https://www.rayyan.ai/), a systematic review and collaboration platform. A standardized data extraction form, developed in Microsoft Excel, was used to facilitate the systematic retrieval of key data points. The form was designed in accordance with academic language and included categories such as study characteristics (e.g., authors, publication year, study design), participant characteristics (e.g., sample size, demographics), intervention details (e.g., rest period strategies, set configuration), outcome measures (e.g., strength, power), and relevant statistical data (e.g., means, standard deviations). Two separate reviewers (F.G. and B.S.) performed the data extraction process using the Rayyan.ai platform, meticulously reviewing and extracting data from each study. Any disagreements or inconsistencies that arose during the process were resolved through consensus reached via comprehensive discussions and data re-examination between both researchers. The data on physical variables were extracted from the text or tables. In cases where the data were presented graphically, an online plot digitizer software (WebPlotDigitalizer, Version 3.12; https://automeris.io/WebPlotDigitizer/) was used to extract the data from the enlarged image.

### Risk of bias analysis

The methodological quality and risk of bias of the included studies were rigorously evaluated using the Cochrane Risk of Bias 2 (RoB 2) tool. This tool is the current gold standard for assessing the internal validity of randomized trials. It evaluates potential sources of bias across five specific domains: (1) randomization process, (2) deviations from intended interventions, (3) missing outcome data, (4) measurement of the outcome, and (5) selection of the reported result. Each domain was rated as ‘low risk’, ‘some concerns’, or ‘high risk’, and an overall risk-of-bias judgment was reached for each study based on these criteria. The risk of bias summary and traffic light plots were generated using the robvis (Risk-of-Bias VISualization) web application [37].

### Meta-analysis

The analysis was carried out using the standardized mean difference (SMDs) and their corresponding 95% confidence intervals (95% CI) as the outcome measure. The interpretation of effect sizes (ES) in all analyses was as follows: < 0.2 for small effects, 0.2–0.6 for moderate effects, 0.6–1.2 for large effects, 1.2-2.0 for very large effects [38]. A positive ES indicated a preference for RR set configurations, while a negative ES favored TS configurations. This situation was accepted only in reverse for the perceived exertion value. The statistical significance threshold was set at *p* < 0.05 for meta-analyses. The mean, standard deviation, and sample size from the RR and TS, respectively, were used to determine the SMD for each outcome. A random-effects model was fitted to the data. The amount of heterogeneity was estimated using the restricted maximum-likelihood estimator [39]. In addition to the estimate of τ^2^, the Q-test for heterogeneity [40] and the I^2^ statistic [41] are reported. Statistical heterogeneity among the studies was assessed using the I² statistic, with values of 25%, 50%, and 75% representing low, moderate, and high heterogeneity, respectively. In case any amount of heterogeneity is detected (i.e., τ^2^ > 0, regardless of the results of the Q-test), a prediction interval for the true outcomes is also provided [42]. Publication bias was assessed using contour-enhanced funnel plots and Egger’s regression test for outcomes with a sufficient number of studies (k ≥ 10). For outcomes with fewer than 10 studies, formal tests for publication bias were not performed due to limited statistical power. Prediction intervals were calculated to estimate the range in which the true effect size of a future study is expected to lie, thereby providing additional information on the dispersion of true effects beyond the confidence interval. The certainty of evidence was evaluated using a simplified GRADE approach, considering Risk of Bias, inconsistency, indirectness, imprecision, and publication bias. The overall certainty of evidence for each outcome was categorized as high, moderate, low, or very low. Perceived exertion was assessed using different scales across studies (e.g., Borg and OMNI scales). To allow comparison and pooling of these outcomes, standardized mean differences (SMD) were used, which account for differences in measurement scales. This approach is commonly recommended when outcomes are conceptually similar but measured using different instruments. The analysis was carried out using R (version 4.3.1) [43], The meta and metaphor package (version 6.2.1) [44].

## Results

### Search results

After conducting a literature review, a total of 1386 studies were identified from PubMed, Web of Science, SPORTDiscus, and Scopus. Following removal of duplicates (*n* = 416), 970 studies remained for screening. Among these, 942 studies were excluded based on the title and abstract. Subsequently, the researchers thoroughly examined the full text of 28 studies. However, 13 studies were ultimately excluded for the following reasons: (a) RR set configuration was not present in four studies; (b) TS configuration was not present in three studies; (c) the design or data of four studies was not appropriate; (d) in two studies, the dependent variables investigated were not relevant to the current research topic. Ultimately, after applying the eligibility criteria, 15 studies were included in the quantitative analysis, as shown in Fig. 1.

### Characteristics of included studies

This meta-analysis includes 15 different studies, encompassing a total of 346 participants with an average age ranging from 20 to 28 years, all of which are summarized in Table 1. The studies employed various resistance training methods such as back squat [19–21, 45–48], bench press [49], knee extension [18, 32], leg press [50], clean pull [51] and countermovement shrug [31]. Intensities are typically determined as a percentage of one-repetition maximum, a specific number of repetitions, or through isokinetic methods. Outcome measures include parameters related to strength, power, and perceived exertion, such as maximal force, mean power, peak power, mean velocity, and rate of perceived exertion (RPE). One of the studies included in the meta-analysis investigated the effectiveness of TS and RR set structures by dividing participants into two different groups based on gender [49]. In their study, Meechan et al. [31], compared the effectiveness of two different RR strategies with the TS set structure. Tufano et al., [18] conducted a comparison of TS and RR methods at two different isokinetic speeds (60°/s and 360°/s). One study, on the other hand, compared the TS and RR set structures at three different percentages of participants’ One-Repetition Maximum (1RM) values (%80, %100, %120) [51]. In the studies included in the research, five [18, 21, 31, 50, 51] used the OMNI scale to determine perceived exertion, while three [19, 45, 46] used the Borg scale. Overall, the included studies predominantly investigated resistance exercises such as squats, bench press, and lower-body movements. The majority of participants were resistance-trained individuals, although some studies included recreationally active participants. Across studies, male participants were more frequently represented, with fewer studies including female or mixed samples. In terms of intervention characteristics, most studies compared traditional set configurations with rest redistribution protocols, with a smaller number examining cluster set variations.

**Table 1:** Summary of included study

Study	Participant			Intervention			Outcomes	
Author, year	MeanAge, Body Mass & Height	Gender	Type & Experience	Set Configuration	Intervention Type	Intervention Intensity	Measures	Results
Boffey et al., 2021 [20]	Men: 22.33 ± 3.45 yrs 80.86 ± 11.39 kg 174.6 ± 6.6 cm Women: 22.20 ± 3.34 yrs 62.23 ± 10.37 kg 160.27 ± 5.89 cm	Mix	Resistance trained Men: 5.02 ± 2.30 yrs Women: 3.60 ± 2.05 yrs	TS: 4 sets of 10 rep, Rest:3 min; RR: 10 sets of 4 rep, Rest: 1 min	Back squat	65% 1RM	-Mean velocity	Mean velocity was significantly higher in RR.
Chae, Hill, et al., 2023 [19]	23 ± 4.8 yrs 78.5 ± 8.6 kg 176 ± 6 cm	Men	Resistance trained 4.4 ± 2.9 yrs	TS: 4 sets of 10 rep, Rest:120-second interset rest; RR: 4 sets of [2 × 5] rep, Rest: 90-second interset rest and 30 s intraset rest	Back squat	70–75% Back Squat 1RM	-Rate of Perceived Exertion	No significant difference between set configurations in RPE.
Jukic &Tufano 2019a [21]	28 ± 5.44 yrs 84.6 ± 10.5 kg (Height not reported)	Men	Strength-trained (Amateur weightlifters and track and field athletes) ≥ 1 yrs	TS: 3 sets of 10 rep, Rest: 4 min; RR: 5 sets of 6 rep, Rest:2 min	Back squat	70% 1RM	- Mean power - Mean power - Rate of Perceived Exertion	Mean velocity and mean power, no significant difference between set configurations, but RPE was less in RR.
Jukic & Tufano 2019b [51]	28 ± 4.48 yrs 89.1 ± 8.7 kg (Height not reported)	Men	Resistance trained ≥ 1 yrs	TS: 3 sets of 6 rep, Rest: 180 s; RR: 9 sets of 2 rep, Intra Rest: 45 s	Clean pull	80% 1RM 100% 1RM 120% 1RM	- Peak power - Mean power	There were small-to-moderate effect sizes in favour of RR80 and RR100, but large effects favouring RR120, compared to them respective TS protocols.
Mayo et al., 2019 [50]	23 ± 2 yrs 68.1 ± 11.9 kg 175 ± 9 cm	Mix	Healthy and moderately trained ≥ 6 months	TS: 5 sets of 8 rep, Rest: 180 s; RR: 1 sets of 40 rep, Rest:18,5 s	Leg press	10 RM	- Rate of perceived exertion	RPE was significantly lower in RR.
Meechan et al., 2023 [31]	27.2 ± 3.3yrs 77.2 ± 10.6 kg 178 ± 7 cm	Men	Various teams and individual sports (national level rugby, soccer, track cycling, martial arts) 7.0 ± 2.2 yrs	TS: 3 set, 6 rep, Rest : 180s min between sets; RR45: 9 set, 2 rep, Rest: 45s between sets; RR72: 6 set, 3 rep, Rest: 72 s between sets	Countermovement shrug	%140 1RM	- Mean force - Mean power - Rate of perceived exertion	No significant difference between set configurations in mean force, mean power and RPE.
Merrigan, Tufano, Oliver, et al. 2020 [47]	23.7 ± 4.1yrs 64.1 ± 10.8 kg 159.8 ± 5.6 cm	Women	Resistance-trained (competitive athletes from strength and collegiate sports) 5.0 ± 2.2 yrs	TS: 4 sets of 10 rep, Rest: 120 s; RR: 4 sets of 10 rep, Intra Set Rest: 30 s, Inter Set Rest:90 s	Back squat	70% 1RM	- Peak power	Peak power was significantly higher in repetitions 7–9 in RR.
Merrigan, Tufano, Fields, et al. 2020 [46]	23.7 ± 4.1yrs 64.1 ± 10.8 kg 160 ± 6 cm	Women	Resistance-trained competitive athletes 5.0 ± 2.2 yrs	TS: 4 sets of 10 rep, Rest: 120 s; RR: 4 sets of 10 rep, Intra Set Rest: 30 s, Inter Set Rest:90 s	Back squat	70% 1RM	-Rate of Perceived Exertion	RPE was significantly lower in RR.
Merrigan, Jones, et al., 2020 [45]	26 ± 1.8 yrs 83.5 ± 9.8 kg 179.1 ± 9.1 cm	Men	Resistance-trained ≥ 6 months	TS: 4 sets of 10 rep, Rest:95 s; RR: 20 sets of 2 rep, Rest: 15 s	Isokinetic unilateral knee extensions	60°/s continuous maximal effort	-Peak torque - Rate of perceived exertion	Peak Torque showed no difference between set configurations, but showed moderate to large effect on repetitions 6,8,10 with higher PT in RR. RPE showed moderate effect in RR.
Merrigan et al., 2022 [32]	26 ± 1.8 yrs 83.5 ± 9.8 kg 179.1 ± 9.1 cm	Men	Resistance-trained ≥ 6 months	TS: 4 sets of 10 rep, Rest:95 s; RR: 20 sets of 2 rep, Rest: 15 s	Eccentric knee extensions	60°/s continuous maximal effort	- Peak torque	No differences between set configurations in peak torque.
Oliver et al., 2016 [22]	27 ± 4 yrs 82.8 ± 6.7 kg 180 ± 7 cm	Men	Resistance-trained highly trained (time not reported)	TS: 4 sets of 10 rep, Rest: 180 s; RR: 4 sets of 10 rep, (30 s intra-rest, 150 s inter-set)	Back squat	70% 1RM	- Mean force - Mean Velocity - Power	Mean power was significantly higher in RR.
Piqueras-Sanchiz et al., 2022 [33]	23.4 ± 4.4 yrs 73.9 ± 9.1 kg 175 ± 5 cm	Men	Resistance-trained ≥ 2 yı	TS: 3 sets of 8 rep, Rest:5 min; RR: 6 sets of 4 rep, Rest: 2 min	Back squat	75% 1RM	- Maximal force	Mechanical performance was significantly higher in RR.
Torrejón et al., 2019 [49]	Men: 23.8 ± 2.5 yrs 73.4 ± 8.9 kg 177 ± 7 cm Women: 21.5 ± 1.4 yrs 62.2 ± 8.7 kg 169 ± 6 cm	Mix	Resistance-trained Men: 6.2 ± 2.0 yrs Women: 1.2 ± 1.5 yrs	TS: 6 sets of 4 rep, Rest: 180 s; RR: 24 sets of 1 rep, Rest:39 s	Bench press	6RM	- Maximal force - Maximal power - Mean Velocity	No statistically significant difference was found between set configurations.
Tufano, Conlon, et al., 2017 [48]	25.2 ± 4.1yrs 76.7 ± 5.1 kg 175 ± 7 cm	Men	Resistance-trained ≥ 6 months	TS: 9 sets of 4 rep, Rest: 52.5 s; RR: 36 sets of 1 rep, Intra Rest: 12 s	Back squat	75% 1RM	- Mean force - Maximal power - Mean power	No interactions or main effects were present in Maximal force. Mean and peak power were maintained at RR1 but decreased across each 4 repeat sequence at TS (RR4).
Tufano et al., 2020 [18]	23 ± 4 yrs 81.06 ± 8.81 kg 181.25 ± 7.34 cm	Men	Resistance-trained ≥ 6 months	TS: 4 sets of 10 rep, Rest: 95 s; RR: 20 sets of 2 rep, Rest: 15 s	Isokinetic knee extension	360° / s	- Peak torque - Mean power - Rate of perceived exertion	RR exhibited higher peak torque and mean power in the last repetitions. Additionally, RPE was lower in RR.

### Assessment of bias

**Fig. 2:**
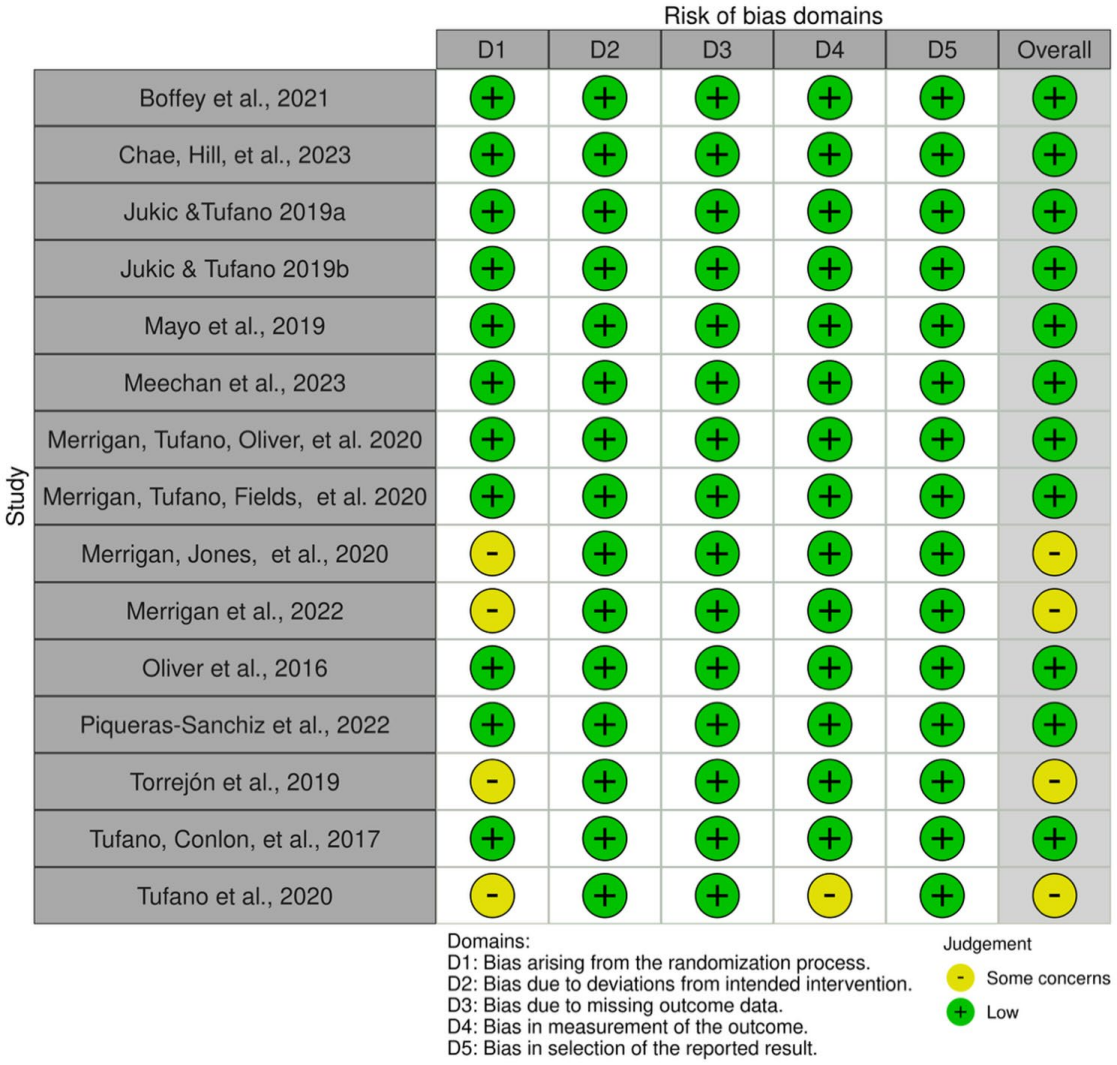
The risk of bias assessment

Based on the RoB 2 assessment, 11 studies (73.3%) were classified as having low risk of bias, while 4 studies (26.7%) were rated as having some concerns. No studies were identified as high risk. These findings indicate that most of the included studies were of acceptable methodological quality; however, the results should be interpreted with caution, particularly in light of the concerns identified in a subset of studies. (Fig. 2).

### Certainty of evidence

The certainty of evidence varied across outcomes. For mean power and peak torque, the certainty of evidence was rated as moderate due to low heterogeneity and consistent findings across studies. For RPE, the certainty of evidence was rated as moderate, although some heterogeneity was observed. For other outcomes such as maximal force, mean force, peak power, and mean velocity, the certainty of evidence was rated as low due to imprecision and variability in effect estimates.

### Meta-analyses results

#### Mean power

**Fig. 3:**
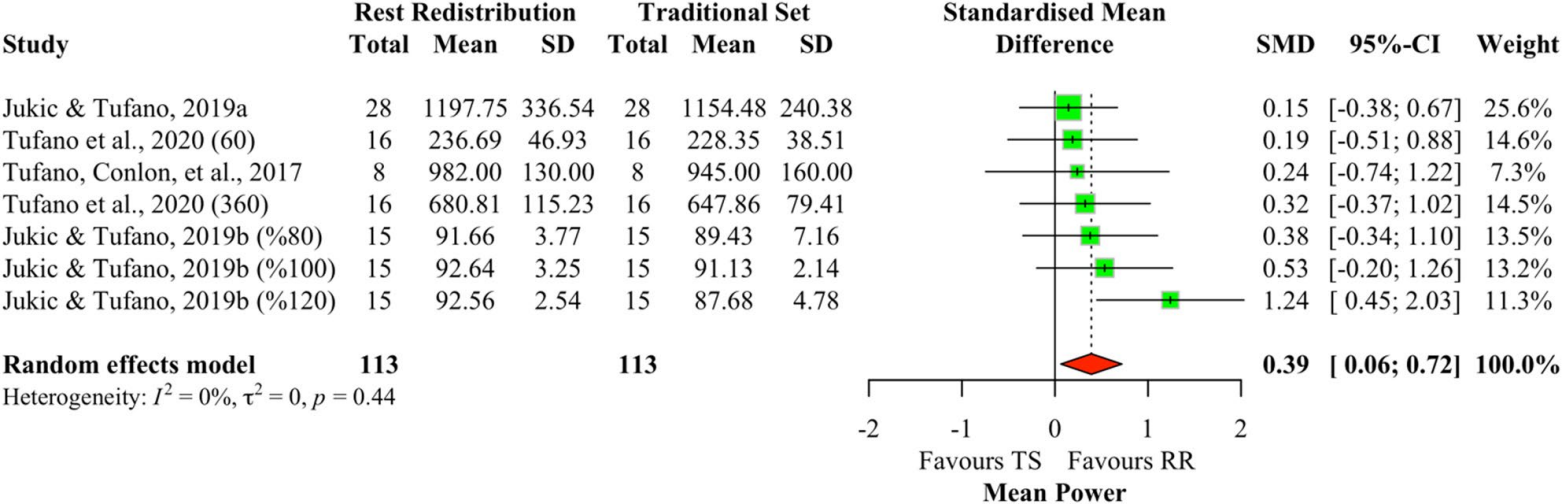
Forest plot showing the observed mean power outcomes

When comparing the impact of TS and RR on mean power, significant effect was found (t = 2.92, *p* = 0.02). The estimated average SMD was 0.39 (95% CI: 0.16 to 0.72). The observed SMDs ranged from − 0.38 to 2.03, with 100% of the estimates being positive. According to the Q-test, there was no significant amount of heterogeneity in the true outcomes (Q(6) = 5.87, *p* = 0.43, τ̂^2 = 0.0, I^2 = 0%). The forest plot showing the relevant data is shown in Fig. 3. A leave-one-out sensitivity analysis was conducted for the mean power outcome. The pooled effect size remained consistent in both magnitude and direction following the exclusion of individual studies, indicating that no single study had a disproportionate influence on the overall results (see Supplementary Figure S1).

#### Peak torque

**Fig. 4:**
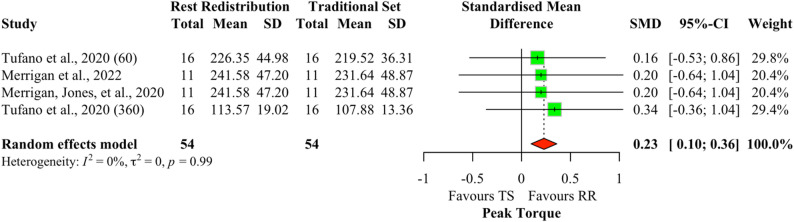
Forest plot showing the observed peak torque outcomes

When comparing the impact of TS and RR on peak torque, significant effect was found (t = 5.54, *p* = 0.01). The estimated average SMD was 0.23 (95% CI: 0.10 to 0.36). The observed SMDs ranged from − 0.53 to 1.04, with 100% of the estimates being positive. According to the Q-test, there was no significant amount of heterogeneity in the true outcomes (Q(3) = 0.14, *p* = 0.99, τ̂^2 = 0.0, I^2 = 0.0%). The forest plot showing the relevant data is shown in Fig. 4. A leave-one-out sensitivity analysis was conducted for the peak torque outcome. The pooled effect size remained stable across all iterations, with no change in the direction or magnitude of the effect, indicating that the results were robust and not influenced by any single study (see Supplementary Figure S2).

#### Rate of perceived exertion

**Fig. 5:**
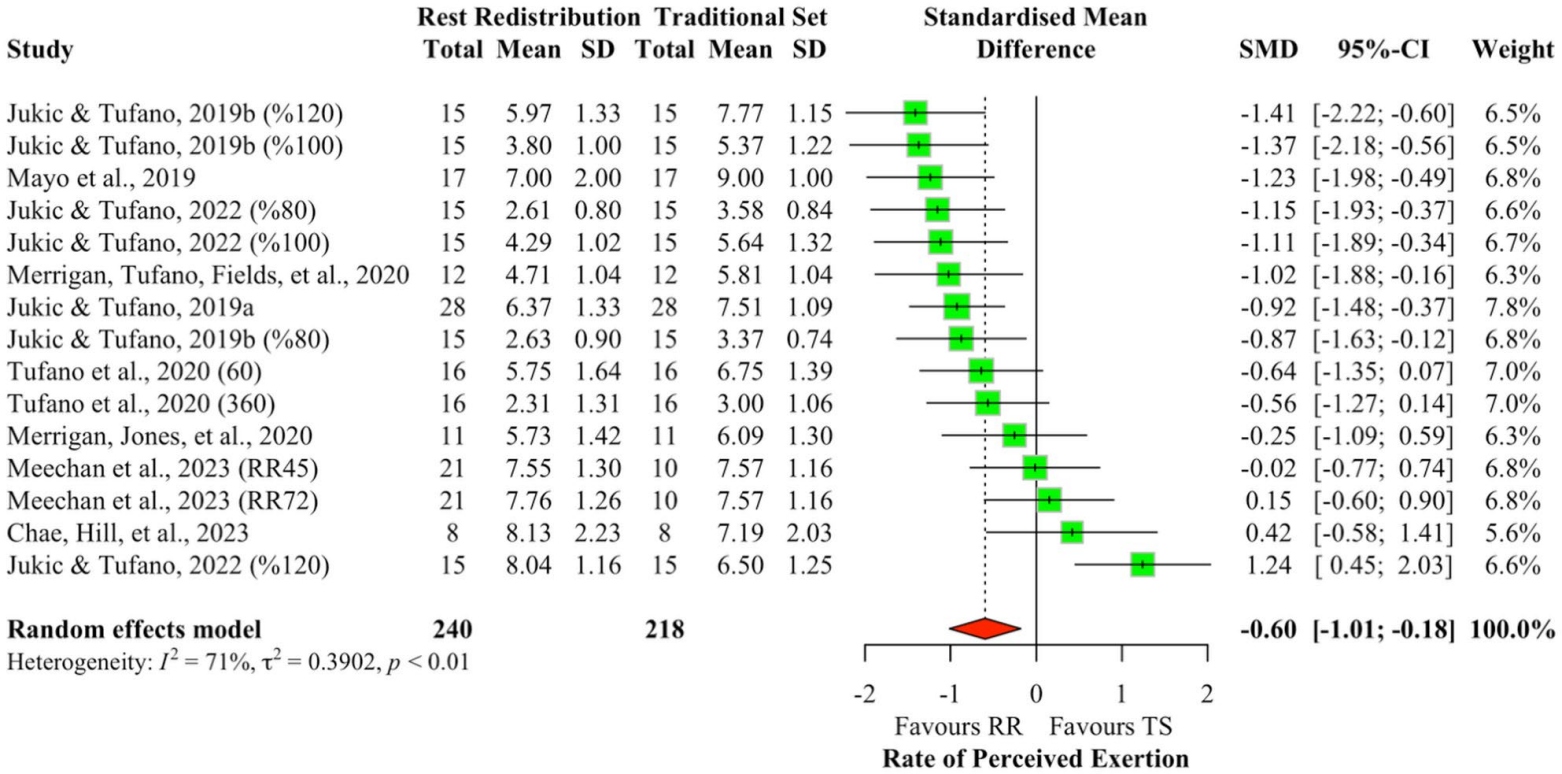
Forest plot showing the observed RPE outcomes

When comparing the impact of TS and RR on RPE, significant effect was found (t = -3.96, *p* = 0.00). The estimated average SMD was − 0.67 (95% CI: -1.04 to -0.30). The observed SMDs ranged from − 2.22 to 1.41, with 83% of the estimates being negative. According to the Q-test, there was significant amount of heterogeneity in the true outcomes (Q(11) = 23.11, *p* = 0.00, τ̂^2 = 0.16, I^2 = 52.4%). The forest plot showing the relevant data is shown in Fig. 5. Publication bias for the RPE outcome was assessed using a contour-enhanced funnel plot and Egger’s regression test. Visual inspection of the funnel plot did not indicate substantial asymmetry. Furthermore, Egger’s regression test showed no significant evidence of publication bias (t = 0.86, *p* = 0.41). A leave-one-out sensitivity analysis was conducted for the RPE outcome to assess the robustness of the pooled effect. The results remained consistent in both magnitude and direction after the exclusion of each individual study, indicating that no single study disproportionately influenced the overall findings (see Supplementary Figure S3).

The prediction interval indicated that the true effect size in future studies may vary across a wider range, suggesting moderate between-study variability.

#### Maximal force

**Fig. 6:**
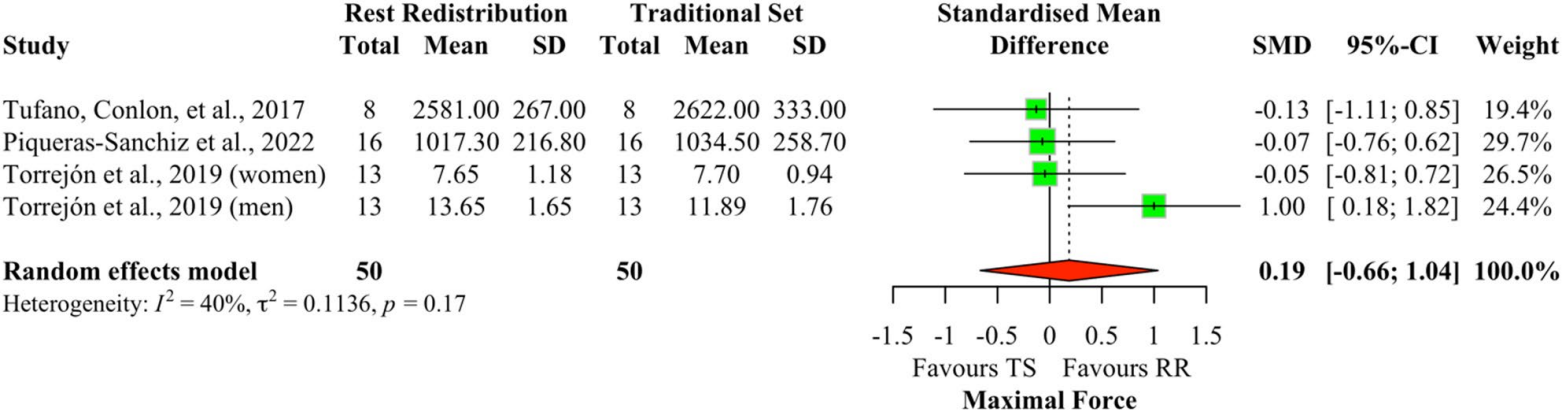
Forest plot showing the observed maximal force outcomes

When comparing the impact of TS and RR on maximal force, no significant effect was found (t = 0.70, *p* = 0.53). The estimated average SMD was 0.19 (95% CI: -0.66 to 1.04). The observed SMDs ranged from − 1.11 to 1.82, with 75% of the estimates being negative. According to the Q-test, there was no significant amount of heterogeneity in the true outcomes (Q(3) = 5.02, *p* = 0.17, τ̂^2 = 0.11, I^2 = 40%). The forest plot showing the relevant data is shown in Fig. 6.

#### Mean force

**Fig. 7:**
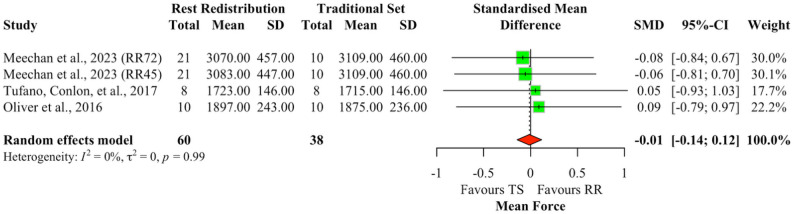
Forest plot showing the observed mean force outcomes

When comparing the impact of TS and RR on mean force, no significant effect was found (t = -0.32, *p* = 0.77). The estimated average SMD was − 0.01 (95% CI: -0.14 to 0.12). The observed Standardized Mean Differences (SMDs) ranged from − 0.84 to 0.97, with 50% of the estimates being positive. According to the Q-test, there was no significant amount of heterogeneity in the true outcomes (Q(3) = 0.11, *p* = 0.99, τ̂^2 = 0.0, I^2 = 0.0%). The forest plot showing the relevant data is shown in Fig. 7.

#### Peak power

**Fig. 8:**
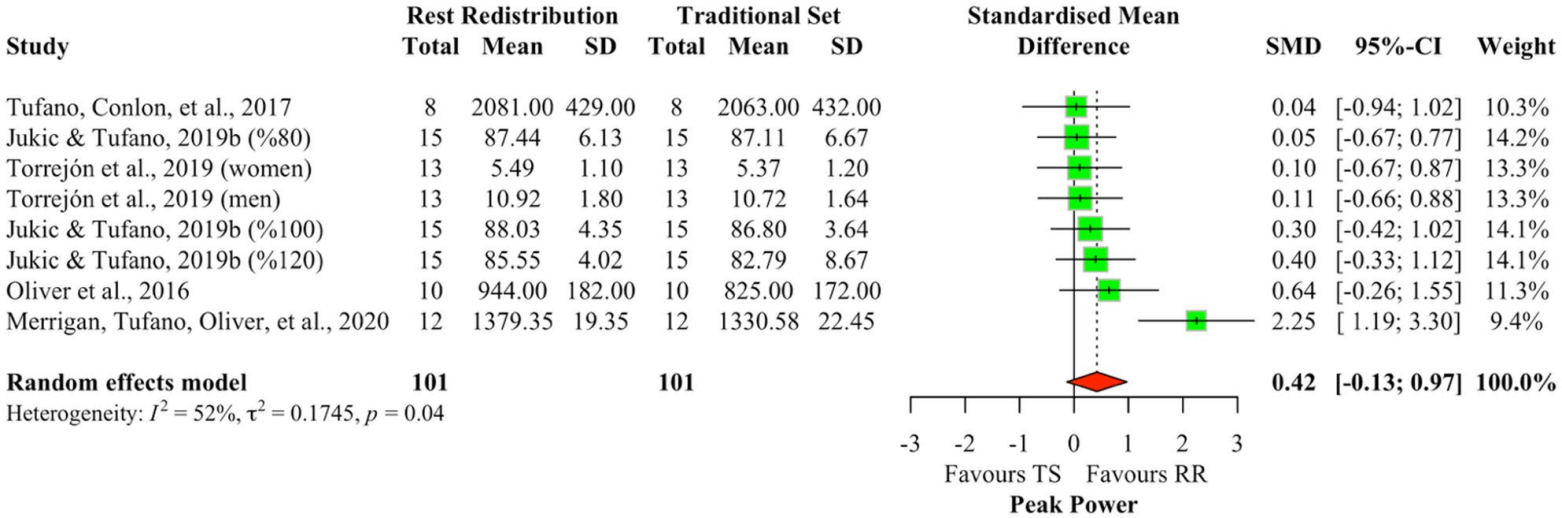
Forest plot showing the observed peak power outcomes

When comparing the impact of TS and RR on peak power, no significant effect was found (t = 1.80, *p* = 0.11). The estimated average SMD was 0.42 (95% CI: -0.13 to 0.97). The observed SMDs ranged from − 0.94 to 3.30, with 100% of the estimates being positive. According to the Q-test, there was significant amount of heterogeneity in the true outcomes (Q(7) = 14.59, *p* = 0.04, τ̂^2 = 0.17, I^2 = 52%). The forest plot showing the relevant data is shown in Fig. 8.

#### Mean velocity

**Fig. 9:**
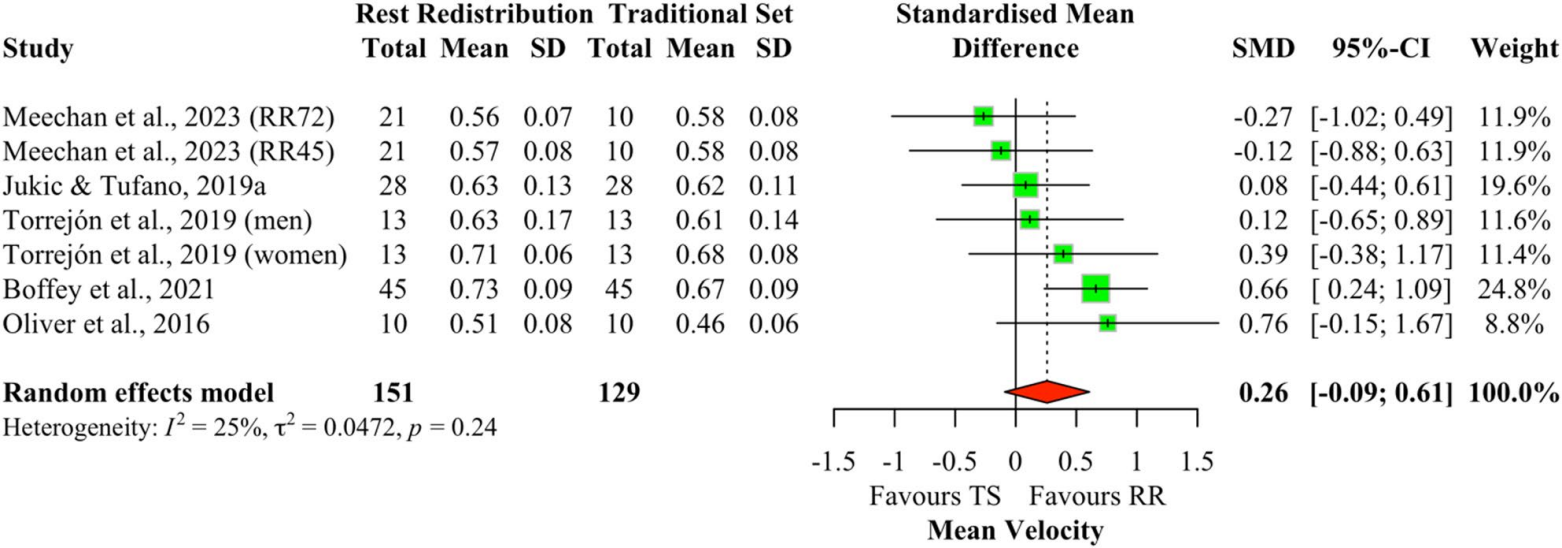
Forest plot showing the observed mean velocity outcomes

**Fig. 10:**
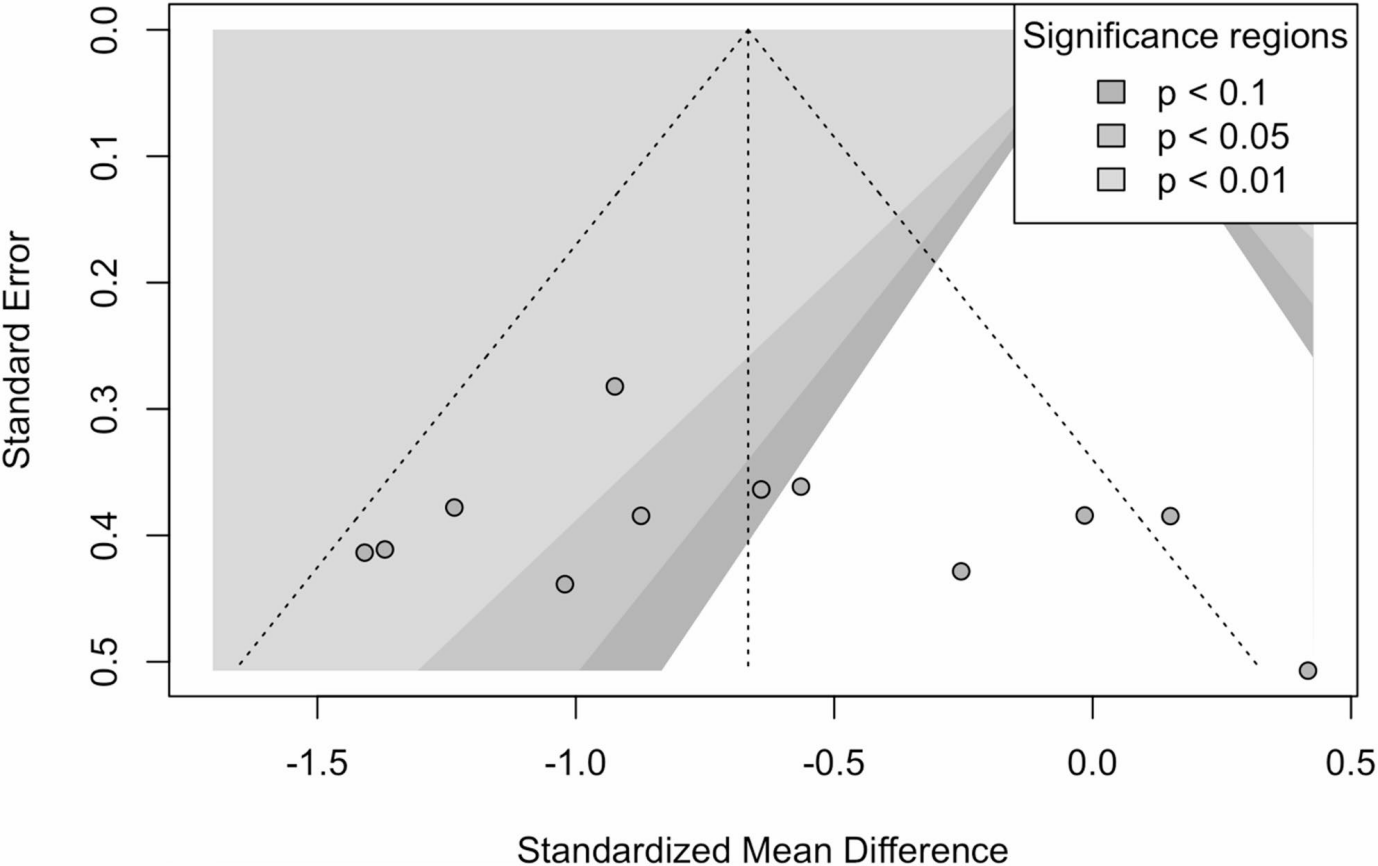
Contour-enhanced funnel plot for the RPE outcome

When comparing the impact of TS and RR on mean velocity, no significant effect was found (t = 1.81, *p* = 0.12). The estimated average SMD was 0.26 (95% CI: -0.09 to 0.61). The observed SMDs ranged from − 1.02 to 1.67, with 71.4% of the estimates being positive. According to the Q-test, there was no significant amount of heterogeneity in the true outcomes (Q(6) = 8.03, *p* = 0.23, τ̂^2 = 0.04, I^2 = 25%). The forest plot showing the relevant data is shown in Figs. 9, 10, Table 2.

**Table 2:** GRADE evidence profile for the effects of rest redistribution versus traditional set configurations on strength, power, and perceived exertion

Outcome	Risk of Bias	Inconsistency	Indirectness	Imprecision	Publication Bias	Certainty of Evidence
Mean Power	Not serious	Not serious	Not serious	Serious^a^	Undetected	Moderate
Peak Torque	Not serious	Not serious	Not serious	Serious^a^	Not assessed	Moderate
RPE	Not serious	Serious^b^	Not serious	Not serious	Undetected	Moderate
Maximal Force	Not serious	Not serious	Not serious	Very Serious^c^	Not assessed	Low
Mean Force	Not serious	Not serious	Not serious	Very Serious^c^	Not assessed	Low
Peak Power	Not serious	Serious^b^	Not serious	Serious^a^	Not assessed	Low
Mean Velocity	Not serious	Not serious	Not serious	Very Serious^c^	Not assessed	Low

^a^Downgraded by one level due to imprecision (limited sample size and/or wide confidence intervals)

^b^Downgraded by one level due to inconsistency (I² > 50%)

^c^Downgraded by two levels due to very serious imprecision (small number of studies and limited total sample size)

## Discussion

This meta-analysis was conducted to compare the effects of TS and RR set structures on strength, power, and perceived exertion during resistance training. When the main findings of the study are examined, it is shown that compared to TS, the RR set structure increases peak torque (*p* = 0.01), and mean power (*p* = 0.02), while reducing rate of perceived exertion (RPE) (*p* = 0.00). Additionally, no statistically significant differences were observed between the two different rest strategies on maximal force (*p* = 0.53), mean force (*p* = 0.77), peak power (*p* = 0.11), and average velocity (*p* = 0.12). Potential sources of heterogeneity may include differences in training protocols, exercise selection, participant characteristics, and loading schemes across studies, which should be considered when interpreting the present findings.

Mean power, as a determining factor in enhancing performance in athletic activities [52], also serves as an indicator of athletes’ sustainable power production capacities, calculated as the area under the upward portion of the power-time curve [53]. When evaluating the findings of this meta-analysis, it is observed that the RR rest strategy during resistance training is moderately more effective than the TS method in maintaining mean power (SMD = 0.39, CI = 0.06 to 0.72). In all studies included in the analysis testing mean power, it was observed that the RR set structure was more effective compared to TS. Only in two studies, the effect level was small, in four studies, it was moderate, and in one study, it was found to have a very high effect level. When the research findings of Jukic & Tufano [51] are examined, it is observed that as the applied load increases during resistance training, the effect of RR rest method on mean power compared to TS increases. Effect values were found to be SMD = 0.38, 0.53, 1.24 when loading was performed with 80%, 100%, and 120% of 1RM, respectively. The RR set configuration, by dividing the total rest time into more frequent intervals, enables the athlete to be exposed to lower volume sets. This can potentially affect fatigue, thereby assisting the athlete in better preserving mean power during resistance training [48]. Using methods focused on improving and preserving mean power during resistance training can optimize performance outcomes in various sports disciplines in the long term.

Peak torque is an important feature in evaluating athletic performance and strength-generating ability. This characteristic demonstrates the athlete’s ability to rapidly generate torque, providing valuable information about power production and overall athletic performance [54]. The current meta-analysis results indicate that RR is a more effective approach compared to TS in terms of peak torque generation during acute resistance training (SMD = 0.23, CI = 0.10 to 0.36). In all studies included in the analysis evaluating Peak Torque, a favorable effect towards TS compared to the RR set structure has been observed. In the study by Tufano et al., [18], a low effect size (SMD = 0.16, CI=-0.53 to 0.86) was observed with isokinetic loading at 60°/s, while a moderate effect size (SMD = 0.34, CI=-0.36 to 1.04) was found with isokinetic loading at 360°/s. Similarly, in two other studies included in the analysis [32, 45], a moderate effect size favoring RR (SMD = 0.20) was observed. Cluster set configurations with longer inter-repetition rest periods have been shown to better preserve barbell technique compared to TSs [55]. The more frequent rest intervals provided in the RR method compared to TS may have led to better preservation of technique and consequently, higher peak torque values obtained. Additionally, Merrigan et al. [45] hypothesized that more frequent rest intervals during resistance training may potentially lead to higher muscle oxygen saturation in active muscles, resulting in higher torque values. When examining the relevant research findings, it is evident that RR has a moderately higher impact on peak torque compared to TS. Therefore, in resistance training where proper technique is crucial, such as for injury prevention, rehabilitation, or with less experienced athletes, opting for RR configurations instead of TS could be considered.

Fatigue can be expressed as a decrease in the ability to maintain optimal muscle strength, leading to performance decline during exercise [56]. In this meta-analysis comparing two different rest methods during resistance training, it is observed that the RR method significantly reduces the perceived level of difficulty compared to the TS method among athletes (SMD = 0.67, CI = -1.04 to -0.30). Overall, the lower perceived level of difficulty under the same load appears to be associated with more frequent rest intervals and consequently, lower volume sets [48]. Fatigue can affect neuromuscular function and thereby lead to a decrease in muscular performance [57]. Additionally, excessive levels of fatigue can increase the risk of injury and impact training progression. In this context, when the goal is to prevent injury risk or avoid the occurrence of acute fatigue, the RR rest method may be a more useful choice for practitioners compared to the TS method. On the other hand, fatigue also affects adaptation to training stimuli and recovery processes. Research has shown that acute fatigue induced by training sessions can contribute to improvements in strength and endurance over time by leading to adaptations in the neuromuscular system [58]. In this context, if the goal of resistance training is to induce acute fatigue on the athlete in order to target chronic adaptations, it may be possible to apply a higher absolute load to the athlete using the RR method compared to the TS method. In their meta-analysis studies investigating the chronic effects of TS and RR, Davies et al. [14] demonstrated that both methods have similar effects on muscle and neuromuscular adaptation. However, they found that the RR rest method induces less fatigue while achieving similar improvements compared to TS. Furthermore, it has been stated in this study that RR can be considered an effective resistance training approach in different stages of periodized programs and various contexts. The findings of our study suggest that RR may be beneficial in individual training sessions due to its acute effects, particularly in fatigue management or during high-volume training periods. Therefore, by using the RR method, there is an opportunity to train with heavier loads at similar levels of fatigue. Athletes and coaches can optimize training programs, enhance performance outcomes, and reduce the risk of overtraining and injuries by effectively monitoring and managing fatigue levels. The RR method can be seen as a valuable tool in terms of set configuration, particularly during periods where fatigue management is critical. However, training using RR exclusively is not necessarily recommended. Instead, its application should be tailored to the specific goals, phases, and needs of the training program.

According to the findings above, when comparing the acute effects of RR and TS methods in this meta-analysis, no significant differences were observed between TS and RR configurations in terms of maximum force (SMD = 0.19, CI = -0.66 to 1.04), mean force (SMD = -0.01, CI = -0.14 to 0.12), peak power (SMD = 0.42, CI = -0.13 to 0.97), and mean velocity (SMD = 0.26, CI = -0.09 to 0.61). These findings suggest that RR may provide advantages in terms of peak torque, mean power, and perceived exertion (RPE) values. However, in the other aspects of resistance training performance examined in this meta-analysis, TS may not offer significant acute benefits. In the study by Davies et al. [14], similar results were obtained in terms of chronic effects. These findings suggest that through the RR method in resistance training, a less fatiguing training experience can be provided under equal volume compared to the TS method, while still achieving similar performance outcomes. This situation could have practical implications that may enhance adherence to resistance training regimes, especially among individuals sensitive to perceived effort levels.

## Limitations

Several limitations of this meta-analysis should be acknowledged. First, the limited number of studies including female participants restricts the generalizability of the findings to women. Second, the applicability of the results to sedentary individuals or clinical populations may be limited, as most included studies involved physically active or resistance-trained individuals. Additionally, the relatively narrow range of training loads examined in some outcomes may limit the interpretation of load-specific effects.

A key limitation of this meta-analysis is the inability to perform subgroup or meta-regression analyses. This was primarily due to the limited number of studies available for several outcomes and the uneven distribution of data across relevant categories such as sex, training status, and type of exercise. Furthermore, variability in training protocols, exercise selection, and participant characteristics reduced the feasibility of conducting meaningful subgroup analyses. Conducting such analyses under these conditions could lead to unstable or misleading results.

Another limitation is the inclusion of different rest-redistribution-related set configurations under a unified framework. Although these approaches share similar underlying principles, they may not be entirely equivalent in their physiological and mechanical effects, potentially contributing to between-study variability. Finally, perceived exertion was assessed using different scales (e.g., Borg and OMNI). Although the use of standardized mean differences enables pooling across measurement tools, differences in scale sensitivity may introduce additional variability.

## Conclusion

This meta-analysis suggests that the RR strategy may offer advantages over TS configurations for certain acute performance outcomes, particularly in increasing mean power and peak torque, and reducing perceived exertion during resistance training. However, these effects were not consistent across all strength and power variables.

Given the moderate-to-low certainty of evidence and the absence of subgroup analyses, these findings should be interpreted with caution. RR should not be considered a universally superior method, but rather a potentially useful strategy that may be integrated into resistance training programs depending on specific goals, contexts, and individual characteristics.

In practical terms, RR may be particularly beneficial during phases where fatigue management and movement quality are prioritized. However, alternative set configurations such as TS or CS may remain appropriate for other training objectives, including hypertrophy and maximal strength development. Further high-quality research is needed to confirm these findings and to better understand the long-term effects of RR across different populations, training statuses, and exercise modalities.

## Supplementary Information

Supplementary Material 1.

Supplementary Material 2.

Supplementary Material 3: Figure S1. Influence analysis (leave-one-out method) for mean power under a random-effects model, demonstrating the stability of the pooled effect size. Figure S2. Influence analysis (leave-one-out method) for peak torque, indicating that the pooled estimates were not driven by any single study. Figure S3. Influence analysis (leave-one-out method) for rate of perceived exertion (RPE), confirming the robustness of the overall effect across studies.

## Data Availability

The datasets used and/or analysed during the current study are available from the corresponding author on reasonable request.
