# Supplementary material for: Comparison of rest redistribution and traditional set configurations in terms of strength, power, and perceived exertion: a systematic review and meta-analysis of randomized trials
Source: BMC Sports Sci Med Rehabil. 2026 Apr 24;18:269. doi: 10.1186/s13102-026-01709-6 (PMC13248243; doi:10.1186/s13102-026-01709-6)
Supplement: Supplementary file 2 — Supplementary Material 2. [file 13102_2026_1709_MOESM2_ESM.pdf]

Database	Search Strategy (Keywords and Strings)	Filters/Limits
**PubMed / MEDLINE**	(("rest redistribution"[Title/Abstract] OR "rest loading"[Title/Abstract] OR "rest interval"[Title/Abstract] OR "rest period"[Title/Abstract] OR "cluster set*"[Title/Abstract] OR "traditional set"[Title/Abstract] OR "set configuration"[Title/Abstract] OR "inter set rest"[Title/Abstract] OR "intra-set rest"[Title/Abstract])) AND (("Resistance Training"[MeSH] OR "power"[Title/Abstract] OR "strength"[Title/Abstract] OR "resistance"[Title/Abstract] OR "force"[Title/Abstract] OR "endurance"[Title/Abstract] OR "exercise"[Title/Abstract] OR "training"[Title/Abstract] OR "weightlifting"[Title/Abstract] OR "fatigue"[Title/Abstract] OR "hypertrophy*"[Title/Abstract] OR "recovery"[Title/Abstract]))	None
**Web of Science**	TS=("rest redistribution*" OR "rest loading" OR "rest interval" OR "rest period" OR "cluster set*" OR "traditional set" OR "set configuration" OR "inter set rest" OR "intra-set rest") AND TS=("power" OR "strength" OR "resistance" OR "force" OR "endurance" OR "exercise" OR "training" OR "weightlifting" OR "fatigue" OR "hypertrophy*" OR "recovery")	None
**Scopus**	TITLE-ABS-KEY(("rest redistribution*" OR "rest loading" OR "rest interval" OR "rest period" OR "cluster set*" OR "traditional set" OR "set configuration" OR "inter set rest" OR "intra-set rest") AND ("power" OR "strength" OR "resistance" OR "force" OR "endurance" OR "exercise" OR "training" OR "weightlifting" OR "fatigue" OR "hypertrophy*" OR "recovery"))	None
**SPORTDiscus (EBSCO)**	TX ("rest redistribution*" OR "rest loading" OR "rest interval" OR "rest period" OR "cluster set*" OR "traditional set" OR "set configuration" OR "inter set rest" OR "intra-set rest") AND TX ("power" OR "strength" OR "resistance" OR "force" OR "endurance" OR "exercise" OR "training" OR "weightlifting" OR "fatigue" OR "hypertrophy*" OR "recovery")	None
