# Supplementary material for: Comparison of rest redistribution and traditional set configurations in terms of strength, power, and perceived exertion: a systematic review and meta-analysis of randomized trials
Source: BMC Sports Sci Med Rehabil. 2026 Apr 24;18:269. doi: 10.1186/s13102-026-01709-6 (PMC13248243; doi:10.1186/s13102-026-01709-6)
Supplement: Supplementary file 3 — Supplementary Material 3: Figure S1. Influence analysis (leave-one-out method) for mean power under a random-effects model, demonstrating the stability of the pooled effect size. Figure S2. Influence analysis (leave-one-out method) for peak torque, indicating that the pooled estimates were not driven by any single study. Figure S3. Influence analysis (leave-one-out method) for rate of perceived exertion (RPE), confirming the robustness of the overall effect across studies. [file 13102_2026_1709_MOESM3_ESM.pdf]

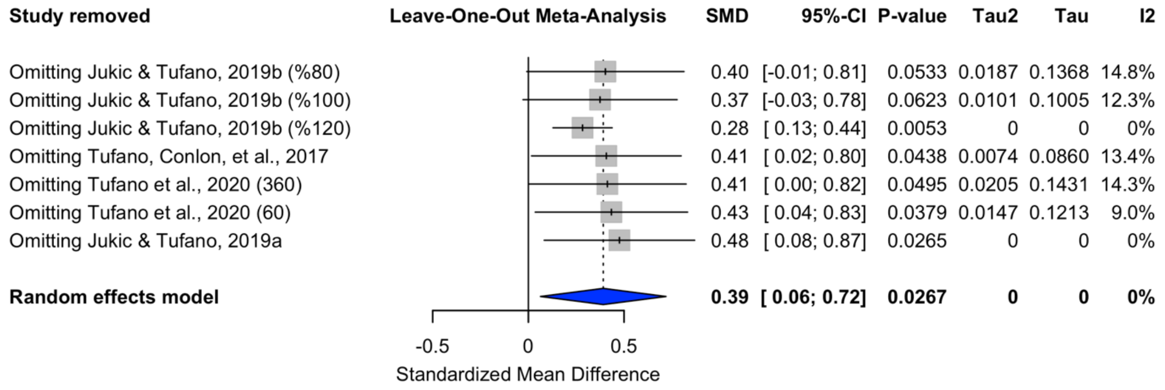

Supplementary Figure S1. Influence analysis (leave-one-out method) for mean power under a random-effects model, demonstrating the stability of the pooled effect size.

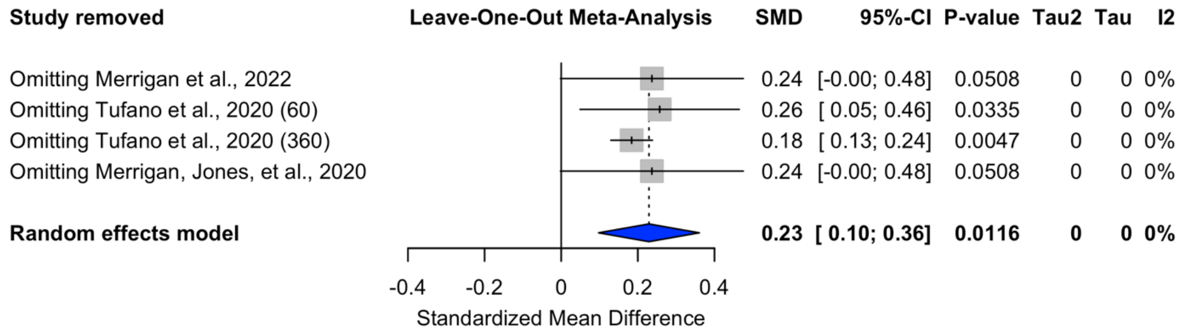

Supplementary Figure S2. Influence analysis (leave-one-out method) for peak torque, indicating that the pooled estimates were not driven by any single study.

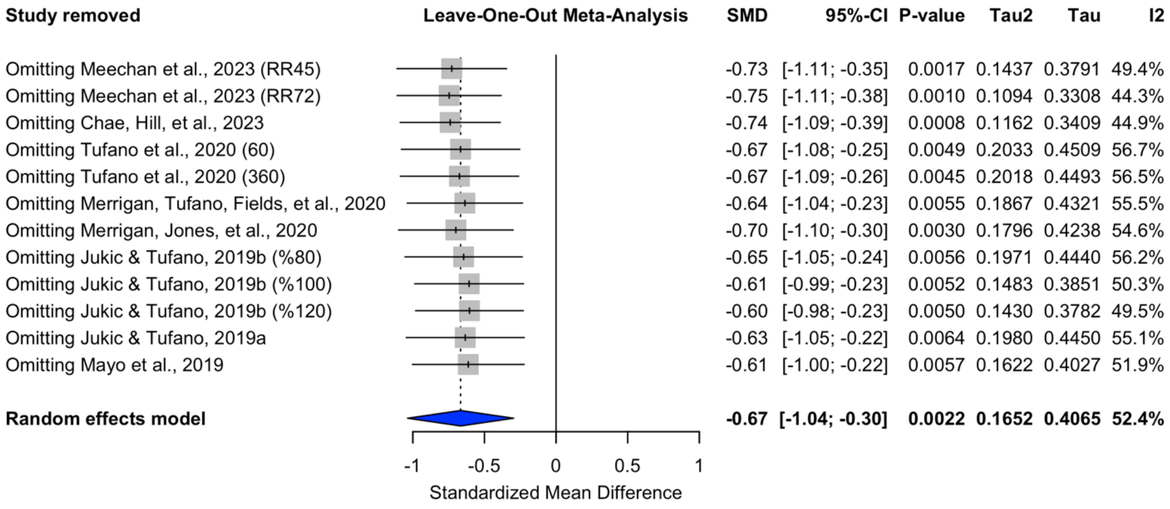

Supplementary Figure S3. Influence analysis (leave-one-out method) for rate of perceived exertion (RPE), confirming the robustness of the overall effect across studies.
